# Optimization of carbon source efficiency for lipid production with the oleaginous yeast *Saitozyma podzolica* DSM 27192 applying automated continuous feeding

**DOI:** 10.1186/s13068-020-01824-7

**Published:** 2020-11-02

**Authors:** Olga Gorte, Michaela Kugel, Katrin Ochsenreither

**Affiliations:** Institute of Process Engineering in Life Science 2: Technical Biology, Karlsruhe Institute of Technology, Fitz-Haber-Weg 4, 76131 Karlsruhe, Germany

**Keywords:** Microbial lipids, Oleaginous yeasts, *Saitozyma podzolica* DSM 27192, Process optimization, Feeding strategy, Continuous feed, Glucose, Xylose, Gluconic acid, Xylonic acid

## Abstract

**Background:**

Biotechnologically produced microbial lipids are of interest as potential alternatives for crude and plant oils. Their lipid profile is similar to plant oils and can therefore be a substitute for the production of biofuels, additives for food and cosmetics industry as well as building blocks for oleochemicals. Commercial microbial lipids production, however, is still not profitable and research on process optimization and cost reduction is required. This study reports on the process optimization using glucose or xylose with the unconventional oleaginous yeast *Saitozyma podzolica* DSM 27192 aiming to reduce the applied carbon source amount without sacrificing lipid productivity.

**Results:**

By optimizing the process parameters temperature and pH, lipid productivity was enhanced by 40%. Thereupon, by establishing a two-phase strategy with an initial batch phase and a subsequent fed-batch phase for lipid production in which a constant sugar concentration of about 10 g/L was maintained, resulted in saving of ~ 41% of total glucose and ~ 26% of total xylose. By performing the automated continuous sugar feed the total sugar uptake was improved to ~ 91% for glucose and ~ 92% for xylose and thus, prevented waste of unused carbon source in the cultivation medium. In addition, reduced glucose cultivation resulted in to 28% higher cell growth and 19% increase of lipid titer. By using xylose, the by-product xylonic acid was identified for the first time as by-product of *S. podzolica*.

**Conclusions:**

These findings provide a broad view of different cultivation process strategies with subsequent comparison and evaluation for lipid production with *S. podzolica.* Additionally, new biotechnological characteristics of this yeast were highlighted regarding the ability to produce valuable organic acids from sustainable and renewable sugars.

## Background

Microbial lipids inhabit a similar lipid profile as plant oils and can be eco-friendly alternatives for several applications, such as biofuels, additives for food and cosmetics industry and building blocks for oleochemicals without competing with food or feed [1–3]. Oleaginous yeasts are one of the most promising microbial lipid factories, since they grow rapidly and are able to produce up to 70% of their cell dry weight (CDW) of lipids [2, 4]. Additionally, for industrial purposes yeast lipid production can be realized in large titers, since the cultivation process is easy to scale-up and independent of season, climate, and location [5, 6]. Neither wide agricultural land is required as it is for oil plants, nor complex light exposure installations as for photoautotrophic microalgae, thus the realization of oleaginous yeasts lipid production is potent to outcompete both the latter.

However, in general these days commercial microbial lipids production is not profitable and only feasible for high-value oils containing high amounts of polyunsaturated fatty acids for nutritional purposes [7–9]. Production of yeast oils with biodiesel profile is still non-competitive with vegetable oils [3, 10, 11]. Costs arise from medium components and preparation, the cultivation process itself and lipid downstream processing as being an intracellular product [2, 3]. More precisely, the carbon source is the main cost factor in the medium and for cost-efficient microbial lipids production as low as possible carbon source amounts and cheap materials are desirable [3]. Processes with high, unused sugar excess in the cultivation broth are uneconomical and not sustainable and should be optimized without sacrificing productivity. Sustainability and economic feasibility for microbial lipids produced by yeasts can be ensured by using abundant and cheap lignocellulosic waste plant biomass as substrate, as it was examined with sugar cane molasses, corncob and other lignocellulosic hydrolysates [12–14].

In this study, we present the cultivation process optimization of the unconventional oleaginous yeast *Saitozyma podzolica* DSM 27192, which was recently screened from soil by Schulze et al. [15]. In addition, Schulze et al. [15] were able to establish a bioreactor cultivation process for lipid production as main product and gluconic acid (GA) as by-product when cultivated on glucose. The process was realized by performing a manual pulsed restock of carbon source to 90 g/L daily, resulting in a steady sugar excess. However, process parameters and conditions were not optimized specifically for *S. podzolica.* The focus of this study, therefore, was first to improve the lipid production potential of the yeast by optimizing the process parameters temperature and pH. Second, precise adjustment of the overall applied sugar amount for optimal needed concentration was examined with the purpose to reduce sugar wastage, since main medium cost are derived from carbon source [3]. In that course, in this study a two-phase process strategy was established comprising a batch cultivation which merges into a fed-batch phase. In the batch phase yeast growth and cell mass production were enabled, whereas by shifting into the fed-batch phase with nitrogen-limiting and sugar-excess conditions, lipid accumulation was stimulated. A two-phase process was often established with other oleaginous yeasts [14, 16–18]. To provide further advance to the cultivation process, manual feeding was replaced by an automated continuous feed in the fed-batch phase. Manual procedures are costly on industrial scale and must be omitted to reduce production costs.

All these cultivation proceedings with *S. podzolica* were optimized on glucose and xylose with the prospect of cultivating this yeast also on sustainable and renewable substrates, such as abundant plant waste material, since the main sugars of lignocellulosic biomass are composed of hexoses and pentoses, such as glucose and xylose [19]. *S. podzolica* showed remarkable abilities to grow on a wide range of other different plant-derived carbohydrates like xylan, inulin and pectin among others [20], which highlights the potential of *S. podzolica* to degrade renewable substrates.

With regard to the organic acids produced as by-products by *S. podzolica,* we are the first to describe *S. podzolica* as xylonic acid (XA) producer by cultivation on xylose. *S. podzolica* was already known to oxidize glucose to GA [15].

Taken together, the aim of this study is to provide an optimized cultivation process of the unconventional oleaginous yeast *S. podzolica* for lipid production on glucose and xylose, whereby it is intended to reduce the applied sugar amount to avoid unsustainable and costly carbon source wastage. Besides that, pulsed and continuous feeding strategies were compared and evaluated. Additionally, the potential of *S. podzolica* to produce valuable organic acids such as GA and XA was further investigated, which in case of the latter was never described before.

## Results

The data shown in this paragraph were obtained either from triplicates (*n* = 3) (shake-flask cultivations) or duplicates (*n* = 2) (bioreactor cultivations).

### Process temperature and pH optimization

The first cultivation process using the oleaginous yeast *S. podzolica* for lipid production was reported by Schulze et al. [15], however cultivation conditions were not optimized. In a shake-flask experiment five different temperatures ranging from 18 to 27 °C were tested in a 96 h cultivation. In Additional file 1: Figure S1, the normalized lipid content per dry cell mass is displayed, revealing 22 °C as the most appropriate temperature at 96 h followed by 20 °C with about 17% less lipid, 25 and 27 °C were about 30% less efficient and 18 °C was the least suitable condition with 40% less produced lipid compared to cultivation at 22 °C.

In the next experimental set-up, optimal pH for lipid production was investigated in bioreactors. Here, all cultivations were performed at 22 °C while pH 4, 5 and 6 was tested. The normalized lipid content over 96 h is presented in Additional file 1: Figure S2 displaying pH 6 as least suitable condition with 50% less lipid at 96 h compared to cultivations with pH 4 or 5, which resulted both in a similar lipid formation.

**Fig. 1:**
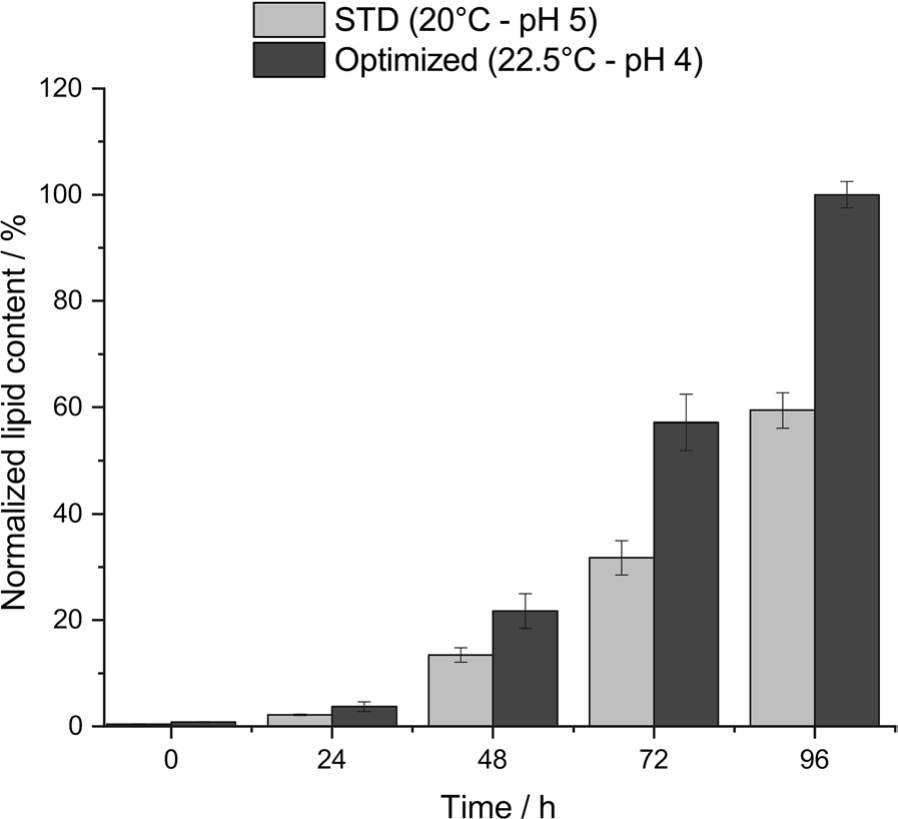
Comparison of produced lipid content by *S. podzolica* within the non-optimized standard process (STD: 20 °C–pH 5) and under temperature and pH optimized conditions (optimized: 22.5 °C–pH 4) in bioreactors over 96-h process time. The data were normalized to the highest lipid titer of 5.98 ± 0.40 g/L. The error bars result from the standard deviation of two independent experiments

To study the individual effects and interactions of influencing factors, a response surface methodology aimed strategy was performed by testing temperatures 20, 22.5 and 25 °C and pH 4; 4.5 and 5 (data not shown). Consequentially, the tendencies of optimal parameters were 22.5 °C and pH 4. To confirm the optimized process parameters, the standard conditions from Schulze et al. [15] (STD: 20 °C; pH 5) were directly compared with the optimized process conditions (Opt: 22.5 °C; pH 4). The normalized lipid content over 96 h of both set-ups is illustrated in Fig. 1. A higher lipid content within the optimized conditions can be observed starting from 24 h, whereby the significance steadily increases over time. At 96 h *S. podzolica* produced 40% more lipid in the optimized process compared to the standard conditions. The highest lipid content produced was 5.98 ± 0.40 g/L.

### Adjustment of applied sugar amount in a two-phase process

**Fig. 2:**
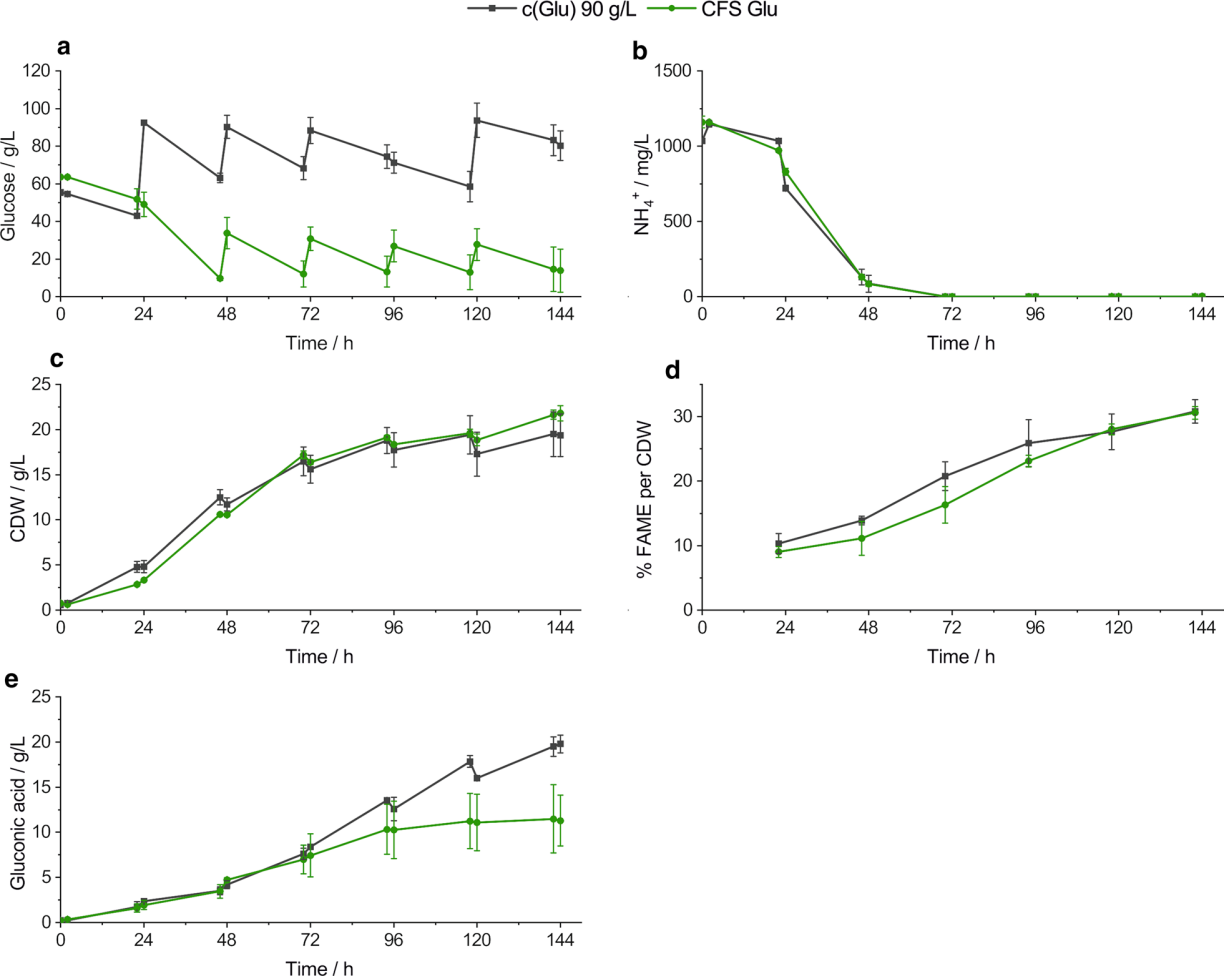
Direct confrontation of daily glucose pulsed feeding cultivation to high sugar excess (c(Glu) 90 g/L) and calculated lower sugar excess feed strategy (CFS Glu). Most important process conditions are illustrated against time. **a** Tracked glucose concentration. **b** Detected ammonia concentration. **c** Cell mass concentration. **d** Analyzed lipid titer. **e** Gluconic acid concentration. The error bars result from the standard deviation of the experimental set-up in duplicates. c(Glu) 90 g/L, standard process with daily glucose restock to 90 g/L; CFS Glu, calculated feed strategy for glucose; CDW, cell dry weight; FAME, fatty acid methyl esters

**Fig. 3:**
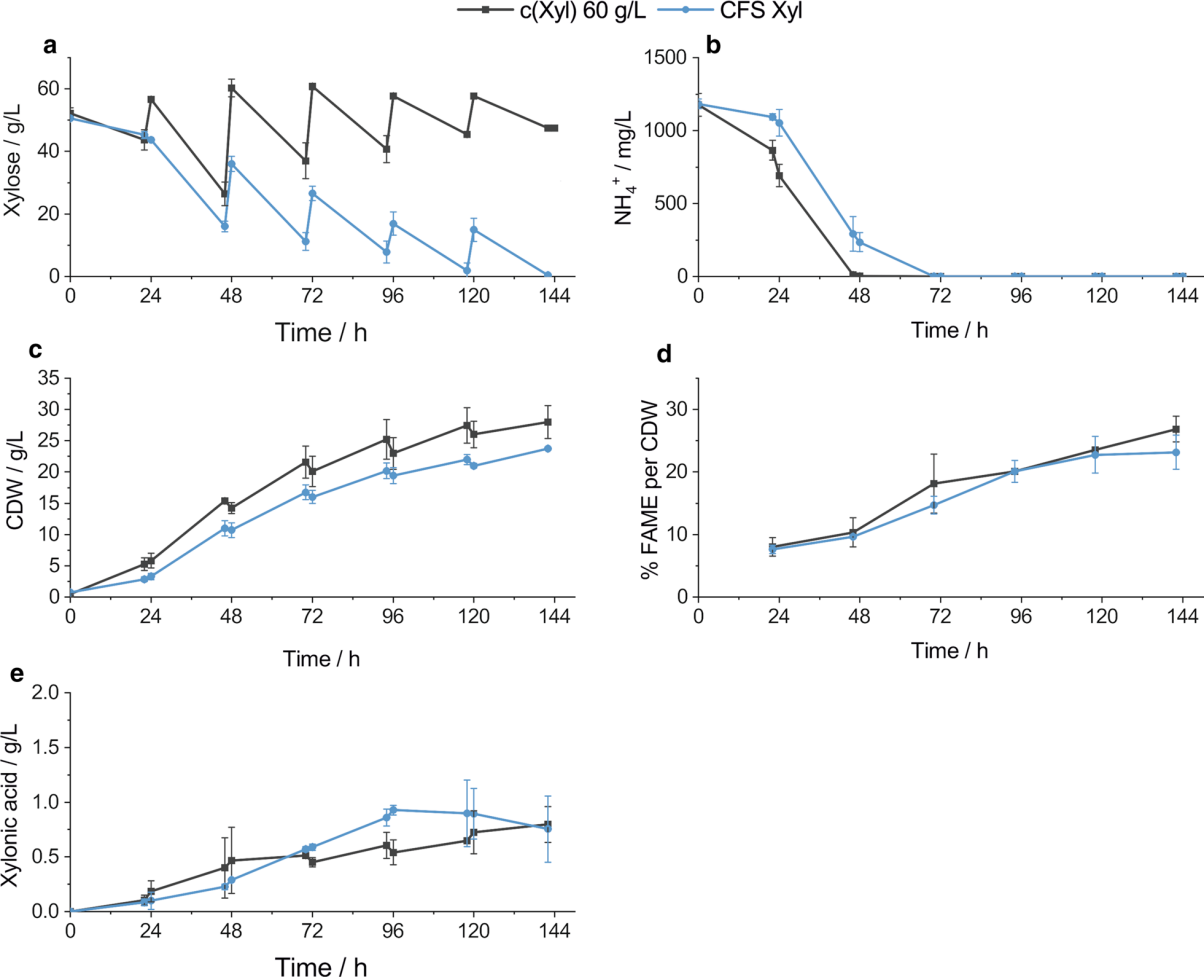
Comparison of cultivations on xylose with daily high xylose excess restock to 60 g/L (c(Xyl) 60 g/L) and calculated lower xylose excess feed strategy (CFS Xyl). Process characteristics like **a** xylose concentration **b** ammonia concentration **c** cell mass concentration **d** lipid titer and **e** xylonic acid concentration, are presented. The standard deviation of the experimental set-up in duplicates is displayed with error bars. c(Xyl) 60 g/L, standard process with daily xylose restock to 60 g/L; CFS Xyl, calculated feed strategy for xylose; CDW, cell dry weight; FAME, fatty acid methyl esters

In the standard cultivation process, as illustrated in Figs. 2 or 3, a large amount of unused carbon source is still present at the end and is therefore wasted. After determining the actual sugar consumption of five independent standard cultivations a new two-phase process comprising an initial batch followed by a daily pulsed fed-batch was developed aiming for a reduced amount of applied sugar.

Figures 2 and 3 display the most important process features of the direct comparison of standard process and adjusted feeding strategy. Figure 2 illustrates the cultivations on glucose as carbon source comparing the daily feeding to 90 g/L (c(Glu) 90 g/L) and the calculated feed strategy (CFS Glu). In Fig. 2a, the two different glucose courses can be observed outlining the change in feeding strategy. The standard process (c(Glu) 90 g/L) starts with an initial glucose concentration of 55.4 ± 1.5 g/L. Every 24 h a manual glucose restock to ~ 90 g/L was performed after determining the amount of consumed sugar, except at 96 h where the feeding procedure was skipped due to failure in glucose monitoring. The average daily glucose concentration before feeding was approximately 65 g/L, i.e., in this process strategy the yeasts were exposed to minimum ~ 65 g/L of sugar excess. The initial glucose concentration in the adjusted process is 63.6 ± 0.5 g/L, which is consumed in the first 46 h in a batch process to 9.6 ± 1.0 g/L. Subsequently, at 48, 72, 96 and 120 h a glucose supply was performed (as reported in Table 4). The average monitored lowest sugar concentration in that process was 12.5 g/L. In total, 269 g glucose was used in the standard process, whereas it was 169 g in the adjusted process resulting in 37.17% less applied sugar. In most of the other process characteristics no significant difference can be observed between both feeding strategies. In Fig. 2b, the ammonia consumption is provided, showing similar consumption behavior in both cultivations. Within the first 48 h most of the ammonia is metabolized and after 70 h ammonia was not detected anymore. The produced cell mass concentration is displayed in Fig. 2c. Both cultivation courses correspond to the ammonia consumption, since in the first 72 h up to 75% of the cell mass was produced. In the latter cultivation period, the cell mass concentration curves flatten. The produced lipids were indirectly analyzed as %FAME per CDW and are shown in Fig. 2d. Here as well, no significant difference can be observed. In the standard process 30.8 ± 1.8% FAME per CDW were produced in the end of the cultivation, whereas with the adjusted feeding strategy 30.6 ± 1.0% FAME per CDW were obtained, respectively. However, by looking at the produced by-product concentration of gluconic acid (Fig. 2e) significantly more was produced at the end of the standard cultivation with 19.9 ± 1.0 g/L compared to 11.3 ± 2.8 g/L from the reduced carbon source feeding.

Equivalent to Figs. 2, 3 presents the process on xylose as carbon source, whereas the daily feeding in the standard process was restocked to 60 g/L (c(Xyl) 60 g/L), which is documented in Fig. 3a. The average daily xylose concentration before feeding was approximately 40 g/L in the standard process. However, the excess xylose concentration increases during the process from 26.4 ± 3.4 g/L at 46 h to 47.4 g/L at 142 h. In the adjusted xylose cultivation process 29.3 g/L xylose were consumed in the first batch phase and at 46 h the xylose concentration was determined to be 16.0 ± 1.7 g/L. A manual xylose supply was performed daily and the added amount is given in Table 4. However, the aimed xylose excess of 10 g/L could not be fully achieved. In the late fed-batch phase at 118 h and 142 h, xylose concentration was determined to be 1.9 ± 2.5 g/L and 0.4 ± 0.3 g/L, respectively. In comparison, 204 g xylose was used for the standard process and 120 g in the reduced carbon source strategy, respectively. In Fig. 3b, c the ammonia consumption and cell mass formation is presented, respectively. The ammonium consumption in the adjusted process was slower compared to the standard process, however, in both cases the ammonia was still exhausted after 70 h (monitored time point). Correspondingly, the cell mass concentration is slightly lower in the adjusted process. Regarding the lipid content (Fig. 3d) no significant difference can be observed. Though the produced lipid did not increase in the adjusted process in the last 24 h. In the standard process 26.9 ± 2.0% FAME per CDW were produced in the end of the cultivation, and 23.2 ± 2.7% FAME per CDW in the adjusted strategy, respectively. As a by-product of the xylose metabolism of *S. podzolica* xylonic acid was identified in the cultivation broth. Opposite to the cultivation on glucose, in both xylose process strategies a similar xylonic acid concentration was produced (Fig. 3e). The maximum amount in the standard process was 0.8 ± 0.2 g/L. In the adjusted process the highest concentration was reached at 96 h with 0.9 ± 0.04 g/L, however, this concentration decreased slightly to 0.8 ± 0.3 g/L.

In Table 1 the cell mass yields (Y_x/s_ [g/g]) and the lipid yields (Y_p/s_ [g/g]) as well as the consumption (Q_s_ [g/L × h]) and production rates (Q_p_ [g/L × h]) are shown for the different cultivation phases. In general, the cell mass yield is higher in the first three cultivation days compared to the latter periods in all monitored processes. This is especially underlined by calculating the yields within the nitrogen metabolism phase (0–72 h) and the N-limitation phase (72–142 h). The cell mass yields in the latter period are about 50% lower. For the lipid yields, however, the tendency is not as clear, but higher lipid yields are shifted to the nitrogen limitation phase.

**Table 1:** Cell mass yields (Y_x/s_ [g/g]), lipid yields (Y_p/s_ [g/g]), consumption (Q_s_ [g/L × h]) and production rates (Q_p_ [g/L × h]) in different cultivation periods in direct comparison between pulsed standard and adjusted pulsed process strategies

Cultivation phase [h]	Y_x/s_ [g/g]				Y_p/s_ [g/g]				Q_s_ [g/L × h]				Q_p_ [g/L × h]			
	c(Glu) 90 g/L	CFS Glu	c(Xyl) 60 g/L	CFS Xyl	c(Glu) 90 g/L	CFS Glu	c(Xyl) 60 g/L	CFS Xyl	c(Glu) 90 g/L	CFS Glu	c(Xyl) 60 g/L	CFS Xyl	c(Glu) 90 g/L	CFS Glu	c(Xyl) 60 g/L	CFS Xyl
0–24	0.34 ± 0.06	0.33 ± 0.11	0.56 ± 0.00*	0.39 ± 0.00*	0.04 ± 0.01	0.04 ± 0.00	0.05 ± 0.01	0.04 ± 0.01	0.57 ± 0.02	0.53 ± 0.22	0.39 ± 0.07	0.24 ± 0.04	0.02 ± 0.01	0.02 ± 0.01	0.02 ± 0.01	0.01 ± 0.00
24–48	0.26 ± 0.02	0.23 ± 0.09	0.32 ± 0.02	0.28 ± 0.01	0.04 ± 0.00	0.04 ± 0.03	0.04 ± 0.01	0.03 ± 0.00	1.33 ± 0.12	1.79 ± 0.25	1.37 ± 0.14	1.26 ± 0.08	0.05 ± 0.00	0.06 ± 0.04	0.05 ± 0.01	0.04 ± 0.01
48–72	0.22 ± 0.04	0.31 ± 0.02	0.31 ± 0.03	0.24 ± 0.00	0.08 ± 0.02	0.09 ± 0.03	0.10 ± 0.02	0.06 ± 0.01	1.00 ± 0.01	0.99 ± 0.06	1.06 ± 0.13	1.13 ± 0.02	0.07 ± 0.02	0.08 ± 0.02	0.09 ± 0.01	0.06 ± 0.01
72–96	0.23 ± 0.01	0.20 ± 0.04	0.26 ± 0.00*	0.22 ± 0.01*	0.10 ± 0.03	0.10 ± 0.02	0.06 ± 0.05	0.08 ± 0.00	0.63 ± 0.04	0.79 ± 0.09	0.91 ± 0.15	0.85 ± 0.06	0.06 ± 0.02	0.07 ± 0.01	0.05 ± 0.05	0.07 ± 0.01
96–120	0.13 ± 0.01	0.15 ± 0.09	0.37 ± 0.03*	0.17 ± 0.02*	0.04 ± 0.00*	0.10 ± 0.00*	0.11 ± 0.01	0.06 ± 0.02	0.58 ± 0.12	0.63 ± 0.04	0.55 ± 0.01	0.68 ± 0.05	0.02 ± 0.00*	0.06 ± 0.01*	0.06 ± 0.00	0.04 ± 0.01
120–144	0.21 ± 0.02	0.22 ± 0.04	0.19 ± 0.05	0.19 ± 0.01	0.05 ± 0.06	0.09 ± 0.02	0.10 ± 0.06	0.03 ± 0.01	0.56 ± 0.05	0.58 ± 0.13	0.43 ± 0.00	0.62 ± 0.15	0.02 ± 0.03	0.05 ± 0.02	0.05 ± 0.03	0.02 ± 0.01
N-metabolization phase	0.25 ± 0.02	0.27 ± 0.07	0.34 ± 0.02	0.28 ± 0.01	0.05 ± 0.01	0.05 ± 0.03	0.06 ± 0.01	0.04 ± 0.00	0.91 ± 0.03	1.04 ± 0.03	0.89 ± 0.06	0.83 ± 0.04	0.05 ± 0.01	0.05 ± 0.02	0.06 ± 0.01	0.04 ± 0.01
N-limitation phase	0.07 ± 0.02	0.12 ± 0.02	0.15 ± 0.01	0.14 ± 0.02	0.06 ± 0.00*	0.10 ± 0.00*	0.09 ± 0.04	0.06 ± 0.01	0.58 ± 0.00	0.61 ± 0.08	0.58 ± 0.04	0.66 ± 0.05	0.04 ± 0.00*	0.06 ± 0.01*	0.05 ± 0.03	0.04 ± 0.00
Total	0.19 ± 0.01	0.22 ± 0.05	0.26 ± 0.01*	0.22 ± 0.00*	0.06 ± 0.00	0.07 ± 0.02	0.07 ± 0.01	0.05 ± 0.01	0.74 ± 0.01	0.83 ± 0.03	0.73 ± 0.05	0.74 ± 0.00	0.04 ± 0.00	0.06 ± 0.02	0.05 ± 0.01	0.04 ± 0.00

Y_x/s_ = cell mass specific yield; Y_p/s_ = product specific yield; Q_s_ = volumetric consumption rate; Q_p_ = volumetric production rate; c(Glu) 90 g/L = standard process with daily glucose restock to 90 g/L; CFS Glu = calculated feed strategy for glucose; c(Xyl) 60 g/L = standard process with daily xylose restock to 60 g/L; CFS Xyl = calculated feed strategy for xylose

* Indicates statistical significant differences (*p* = 0.05)

The highest sugar consumption rates were detected on the second cultivation day in all processes with 1.33 ± 0.12 g/(L × h) in the standard process on glucose and 1.79 ± 0.25 g/(L × h) in the CFS on glucose. On xylose the rates were 1.37 ± 0.14 g/(L × h) in the c(Xyl) 60 g/L process and 1.26 ± 0.08 g/(L × h) in the xylose adjusted strategy. Similar to the lipid yields, the lipid production rates show no clear tendencies.

To illustrate, that despite the sugar reduction of the calculated feed strategy (CFS) process, productivities remained comparable to the standard process, of each phase a mean comparison analysis of standard process against the adjusted process was performed. No significant differences were observed in the majority of the cases. Just in a few periods statistical differences at level *p* = 0.05 were detected, which is within the cell mass yield on xylose at the first, fourth, fifth cultivation day and for the total yield. The lipid yield on glucose is significantly different between 96 and 120 h and in the N-limitation phase. Correspondingly, in the same phases the production rates on glucose are significantly different.

### Automated continuous sugar feed

**Fig. 4:**
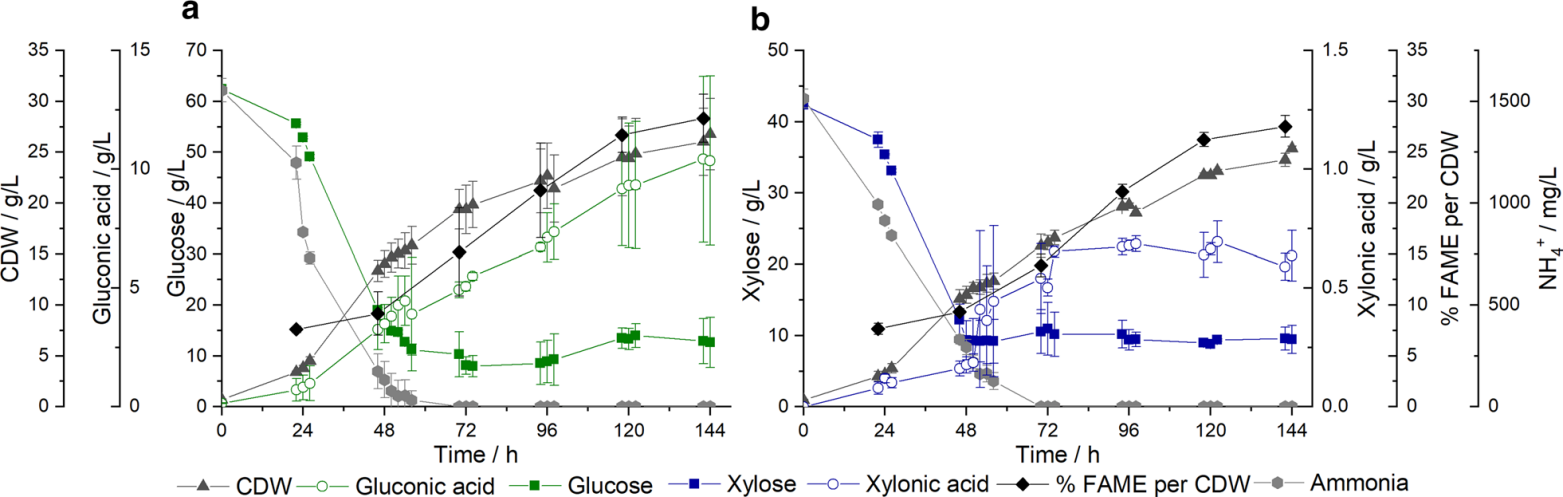
Process plots of established automated continuous feeding with constant sugar excess of about 10 g/L in the fed-batch phase on **a** glucose and **b** xylose. Error bars result from the standard deviation of the experimental set-up in duplicates. CDW, cell dry weight; FAME, fatty acid methyl esters

In the next step, it was intended to omit the manual pulsed feeding in the fed-batch phase and to establish a continuous feeding with constant carbon source excess concentration of about 10 g/L. In Fig. 4 the resulting cultivation plots on glucose (Fig. 4a) and xylose (Fig. 4b) are presented.

In the first 48 h of batch process on glucose (Fig. 4a) 46.21 ± 0.24 g/L glucose was consumed. In the following 96 h of continuous feed the average detected glucose concentration was 11.54 ± 2.28 g/L. As in the pulsed feeding processes, ammonia was completely metabolized after 70 h (monitored time point). At that time point the cell mass concentration reached 19.38 ± 1.94 g/L and increased to 26.78 ± 3.50 g/L till the end of cultivation. As for the products, the maximum content of lipids was measured at the end of cultivation with 28.34 ± 2.38% FAME per CDW, likewise, the maximum titer of gluconic acid was detected at 10.42 ± 3.50 g/L at 142 h. For the cultivation of xylose, the sugar consumption of 33.91 ± 2.64 g/L is illustrated in the first 48-h batch phase in Fig. 4b. Subsequently, a constant xylose concentration of 9.59 ± 0.60 g/L (average of monitored sampling points) was ensured during continuous feeding. Ammonia was exhausted at 70 h. The highest cell mass concentration and lipid content were reached at the end of cultivation with 25.38 ± 0.25 g/L CDW and 27.53 ± 1.07% FAME per CDW, respectively. The detected xylonic acid concentration was slightly lower compared to the manual fed-batch. As shown in Fig. 4b, the maximum concentration was reached at 122 h with 0.70 ± 0.09 g/L, but it decreased to 0.63 ± 0.11 g/L at the end of the cultivation.

### Comparison of process characteristics on glucose and xylose

**Fig. 5:**
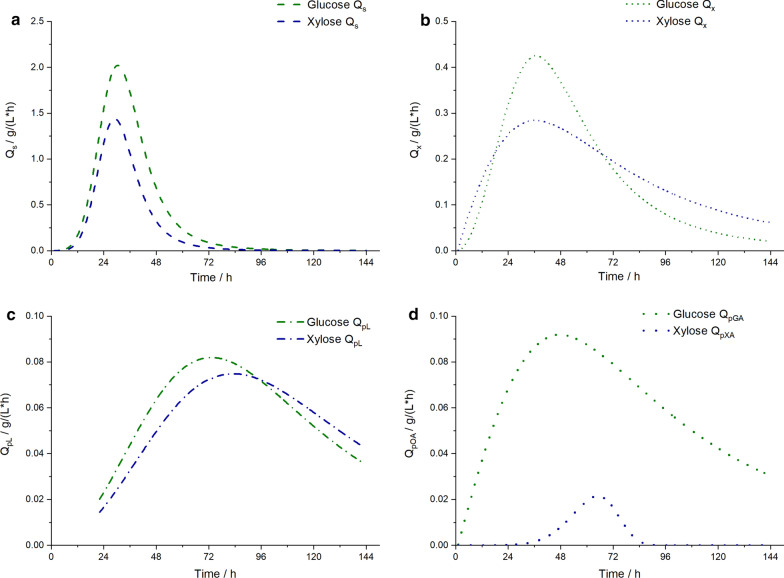
Volumetric consumption and production rates Q [g/L × h] of *S. podzolica* from automated continuous feed process. Q was calculated as derivation of logistic curve fits of carbon source, cell mass, lipid and gluconic or xylonic acid, respectively, on glucose and xylose against time. **a** Q_s_ volumetric substrate consumption rate. **b** Q_x_ volumetric cell mass production rate. **c** Q_pL_ volumetric lipid production rate. **d** Q_pOA_ volumetric organic acid production rate, in detail Q_pGA_: volumetric gluconic acid production rate, Q_pXA_: volumetric xylonic acid production rate

To highlight the differences of *S. podzolica*’s process behavior on continuous feed of glucose versus xylose, volumetric consumption and production rates (Q [g/L × h]) were calculated after logistic curve fits of carbon source, cell mass, lipid and gluconic and xylonic acid, respectively. The comparison is presented in Fig. 5. The volumetric sugar consumption rates are illustrated in Fig. 5a. The peak of the curves reflects the highest rate reached and it can be clearly seen, that on glucose the productivity is more efficient with 2.02 g/(L × h) at 29.8–31 h compared to xylose with 1.44 g/(L × h) at 28.4–29.5 h. In addition, the parabolic curve of glucose consumption is wider and flattens only at 70.3 h below 0.1 g/(L × h), whereas the same occurs 10.9 h earlier at 59.4 h on xylose. The cell mass production rates in Fig. 5b differ greatly. The cell mass production on glucose reaches higher productivity rates with 0.43 g/(L × h) at 36.0–37.3 h at its peak, and on xylose at maximum 0.28 g/(L × h) at 29.5–44.3 h. However, the cell mass productivity on glucose drops faster compared to the cell mass production on xylose, where the curve is at its peak for 14.8 h. At the end of the cultivation the cell mass productivity rate on xylose (0.062 g/(L × h)) is still threefold higher compared to glucose (0.021 g/(L × h)). The volumetric productivity rates of lipids (Fig. 5c) and gluconic or xylonic acid (Fig. 5d), respectively, vary as well. The lipid productivity on glucose is higher at the peak (0.082 g/(L × h) between 56.9 and 65.6 h) than on xylose (0.075 g/(L × h) between 68.3 and 77.5 h), though from 87.5 h the lipid productivity on xylose is higher than on glucose. Most significantly the organic acid productivity curves differ between the two different sugars. Gluconic acid volumetric productivity reaches at maximum 0.092 g/(L × h) between 44.4 and 51.0 h, whereas xylonic acid productivity is maximally achieved between 61.7 and 68.0 h with 0.021 g/(L × h), which is 4.4 times lower compared to gluconic acid productivity.

**Fig. 6:**
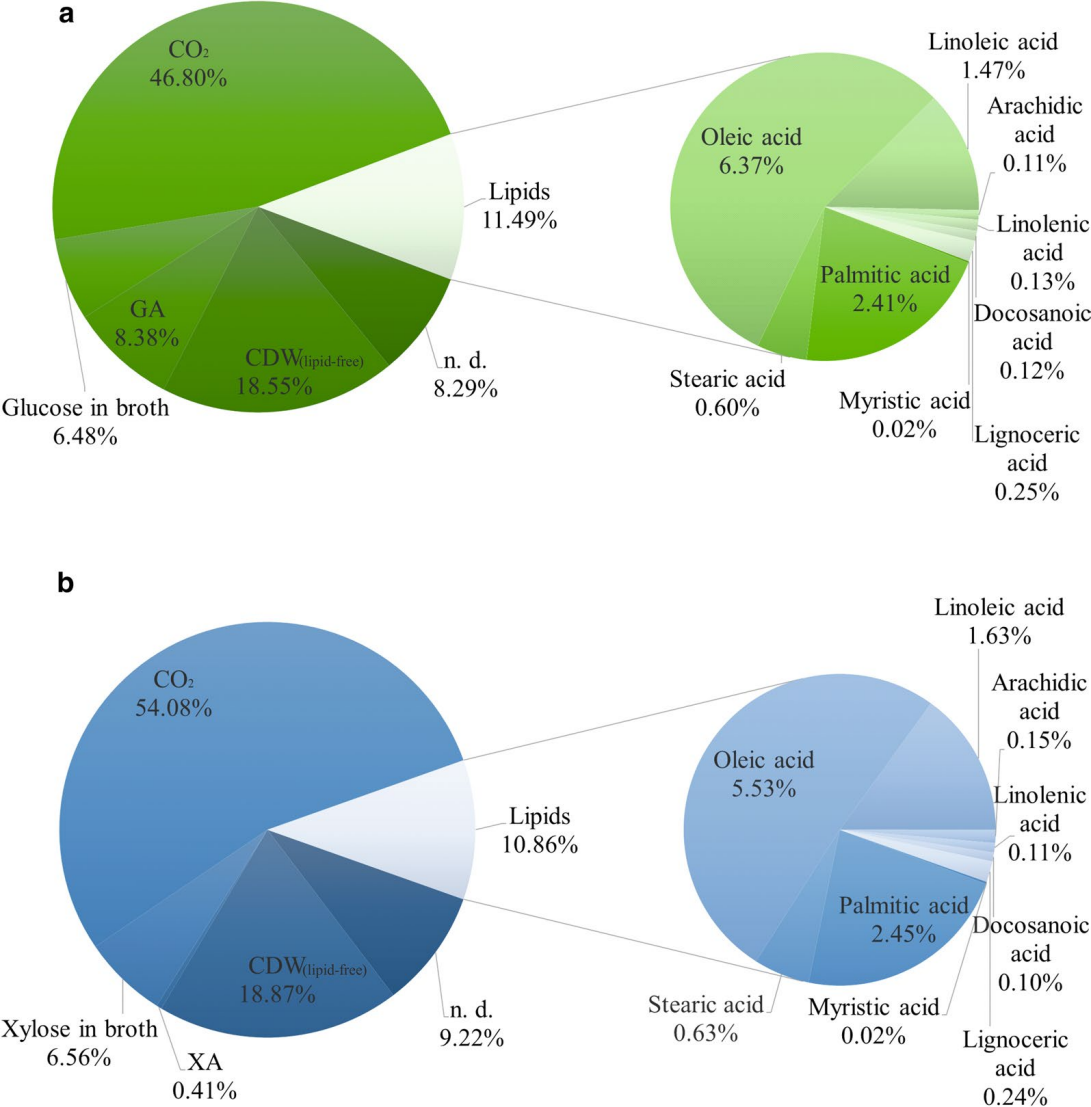
Approximative determination of the overall carbon distribution to process products including by exhaust gas assessed CO_2_ emission from automated continuous feed process of *S. podzolica*. **a** Approximative percentage distribution of glucose conversion. **b** Approximative percentage distribution of xylose conversion. CDW, cell dry weight; n.d., not determined

Besides comparing the production rates, it was also aimed to approximate the overall carbon conversion of both sugar sources by assessing the exhaust gas and the analyzed products during continuous feeding process. The plots of the CO_2_ emission over time are provided in Additional file 1: Figure S3a for glucose and Additional file 1: Figure S3c for xylose. Here, a difference of CO_2_ production curves on glucose or xylose can be observed. While on glucose a peak of produced CO_2_ is reached during initial batch phase, which later decreases, on xylose beginning from the second cultivation day a nearly constant CO_2_ production was detected. The approximate carbon balance is shown in Fig. 6 on glucose (Fig. 6a) as well as on xylose (Fig. 6b) a similar percentage of the overall carbon was converted into lipid-free cell mass (glucose: 18.55%; xylose: 18.87%) and lipids (glucose: 11.49%; xylose: 10.86%). However, on glucose clearly more carbon is channelled to produce the organic acid gluconic acid (8.38% of total carbon). Correspondingly, less CO_2_ was produced on glucose with 46.8% of total carbon, on xylose, however, is was 54.08%. Regarding the lipids, on both carbon sources a similar lipid profile ratio can be observed with oleic acid as main fatty acid followed by palmitic, linoleic and stearic acid. The amount of unconverted carbon, left in the cultivation broth, was comparable in both cases.

## Discussion

### Influence of temperature and pH on lipid production

The first cultivation process using the oleaginous yeast *S. podzolica* for lipid production was reported by Schulze et al. [15], however cultivation conditions (temperature: 20 °C, pH 5) were not optimized. In order to reach the maximum potential of the production stain, in this study these process parameters were investigated to find the optimal conditions. According to literature, for oleaginous yeast temperature should generally be maintained between 25 and 30 °C, while pH is to be regulated between 3 and 6 [6]. However, an optimal lipid production temperature for the unconventional oleaginous yeast *S. podzolica* is barely known in literature. Here rather functional cultivation conditions at 25 or 30 °C can be found to obtain cell mass for several characterization studies [21, 22]. As an isolate from peat bog [15], known as acidic soil, acidic pH (4–6) and temperate temperatures between 18 and 27 °C were considered to test. Interestingly, the warm-moderate temperatures (25 and 27 °C) were unsuitable for the lipid productivity of *S. podzolica.* Still less suitable was the cold-moderate temperature of 18 °C for lipid production (Additional file 1: Figure S1). As expected, a more acidic pH range turned out to be more suitable compared to pH 6 (Additional file 1: Figure S2). After a response surface aimed methodology (data not shown) the tendencies of optimal parameters were directed to 22.5 °C and pH 4. Since basic knowledge about carbon source and sugar concentration [20, 23] was present, this response surface methodology dealt with yet unknown parameters, namely temperature, pH and agitation. For agitation it was observed that the 600 rpm of the standard process were already optimal. The direct comparison of non-optimized standard process from Schulze et al. [15] versus the optimized conditions revealed a 40% increase of produced lipids at 96 h, as shown in Fig. 1. Thus, a remarkable impact of temperature and pH on the lipid content of *S. podzolica* can be concluded. In similar studies of other oleaginous yeasts the influence of temperature and pH, among other, was also reported [24–27].

### Reduction of excess carbon source amount in a two-phase cultivation process

To optimize the cultivation process not only in the aspect of productivity, but also in an economical context, a reduction of used sugar amount was sought while still ensuring a comparable lipid content. Since the accumulation of triacylglycerols (TAGs), as intracellular storage lipids in the yeast cell, is due to a stress response by lacking nutrients, e.g., nitrogen, with simultaneous excess of carbon source, high C/N ratios are required for lipid production [4, 9]. The optimum C/N ratio is reported to be close to 100 for most of the oleaginous yeasts [2, 5, 6]. However, too early or unregulated nitrogen limitation will impact the yeasts growth and deteriorate the cell mass production and consequently the SCO titer, as being an intracellular product [28]. For that reason, in this study a two-phase process was introduced, comprising an initial 48-h batch cultivation without nutrient limitation for the purpose of cell mass production and merging into a pulsed (Figs. 2 and 3) or continuous (Fig. 4) fed-batch process, respectively, with nitrogen-limiting conditions. The initial C/N ratio in all in this study established cultivations was always kept high (glucose:130; xylose: 90). However, it was observed that in the fed-batch phase, where nitrogen is limited, the sugar excess can be reduced and does not need to be restocked to 90 g/L (glucose) or 60 g/L (xylose), respectively, as it was performed in the non-optimized standard process. As proven in Table 1, yields and productivities of *S. podzolica* on both high and reduced carbon source cultivations were similar. On glucose even an improvement in cell mass or product yields and productivities, respectively, can be observed. Too high sugar concentrations are not only uneconomical, but can rather lead to narrow productivity, due to substrate inhibition. It is known, however, that *S. podzolica* is able to tolerate high glucose concentrations of up to 150 g/L for lipid production, although inhibiting effects on cell propagation were observed, which consequently decreased the lipid titer [23]. Therefore, resulting from this study, for lipid production with *S. podzolica* high C/N ratio in the batch phase is mandatory, however, once nitrogen is limited the excess sugar concentration of 10 g/L ensured stable productivities, as can be seen in Fig. 4. Fei et al. [14] were also able to establish an automated system, where the glucose concentration of a lignocellulosic hydrolysate was regulated around 10 ± 2 g/L, and led to a 52% higher lipid content and a 42% lipid productivity improvement compared to a batch cultivation.

However, as can be seen from this study’s Fig. 3a on the calculated feed strategy on xylose (CFS Xyl), the desired concentration of 10 g/L xylose could not be maintained constantly. In the last two cultivation days the lowest xylose concentration reached 1.9 ± 2.5 g/L at 118 h and 0.4 ± 0.3 g/L at 142 h, which led to a stagnation of lipid production (Fig. 3d). Consequently, it can be concluded that at lower sugar concentrations the lipid production decreases and it can be assumed that a constant concentration of 1 g/L might be too low and should be avoided for stable productivities.

Thus, in the reduced sugar cultivation with constant feeding and regulated sugar excess of about 10 g/L 40.52% and 26.47% of glucose or xylose, respectively, were saved compared to the un-optimized standard process. By comparing the actual metabolized sugar with the applied sugar, the difference between optimized and un-optimized feeding strategies is remarkable. The process of daily glucose restocking to 90 g/L led to a sugar utilization of just 56.01 ± 4.35%, in comparison to 90.84 ± 3.78% within the optimized constant feeding (Table 2). On xylose 72.49 ± 0.56% of sugar were metabolized in the daily restocking to 60 g/L strategy, but 92.30 ± 1.61 in the constant feeding (Table 2). However, the total metabolized sugar amount does not differ significantly between the feeding strategies (Table 2). Hence, the constant feed reduced sugar strategy led to a more efficient carbon source uptake in the cultivation process and prevented wastage of unused sugar in the broth.

**Table 2:** Comparison of high sugar standard cultivation and reduced sugar cultivation with continuous feed

	c(Glu) 90 g/L	Cont. CFS Glu	c(Xyl) 60 g/L	Cont. CFS Xyl
Feeding strategy	Daily manual restock	Batch/continuous fed-batch	Daily manual restock	Batch/continuous fed-batch
Feeding status	Un-optimized	Optimized	Un-optimized	Optimized
Total sugar amount applied [g]	269	160	204	150
Total sugar amount metabolized [g]	150.66 ± 11.71	145.35 ± 6.05	147.88 ± 1.17	138.46 ± 2.40
% sugar used by yeasts	56.01 ± 4.35	90.84 ± 3.78	72.49 ± 0.56	92.30 ± 1.61
Final CDW [g/L]	19.38 ± 2.37	26.78 ± 3.5	27.98 ± 2.65	25.38 ± 0.23
Final lipid content [% FAME/CDW]	30.82 ± 1.84	28.34 ± 2.38	26.87 ± 2.04	27.53 ± 1.07
Final lipid titer [g/L]	5.98 ± 0.40	7.41 ± 1.55	7.54 ± 1.28	6.68 ± 0.43
Final organic acid concentration [g/L]	GA: 19.78 ± 0.98	GA: 10.38 ± 3.57	XA: 0.90 ± 0.16	XA: 0.64 ± 0.11
Total consumption rate [g/(L × h)]	0.71 ± 0.06	0.88 ± 0.06	0.87 ± 0.03	0.78 ± 0.01
Total cell mass production rate [g/(L × h)]	0.14 ± 0.02	0.19 ± 0.02	0.20 ± 0.02	0.18 ± 0.02
Total lipid production rate [g/(L × h)]	0.04 ± 0.00	0.05 ± 0.01	0.05 ± 0.01	0.05 ± 0.00
Total organic acid production rate [g/(L × h)]	GA: 0.14 ± 0.01	GA: 0.07 ± 0.03	XA: 0.006 ± 0.001	XA: 0.0004 ± 0.0001
Total Y_x/s_ [g/g]	0.19 ± 0.01	0.21 ± 0.01	0.26 ± 0.01	0.22 ± 0.00
Total Y_l/s_ [g/g]	0.06 ± 0.00	0.06 ± 0.01	0.07 ± 0.01	0.06 ± 0.00
Total produced CO_2_ [g]	84.62	109.79	84.96	118.90
Total produced CO_2_ per CDW [g/g]	2.98 ± 0.36	3.58 ± 0.38	2.42 ± 0.05	3.83 ± 0.04

c(Glu) 90 g/L = standard process with daily glucose restock to 90 g/L; Cont. CFS Glu = calculated feed strategy for glucose with continuous feed; c(Xyl) 60 g/L =  standard process with daily xylose restock to 60 g/L; Cont. CFS Xyl = calculated feed strategy for xylose with continuous feed, Y_x/s_ = cell mass specific yield; Y_l/s_ = lipid specific yield, CDW = cell dry weight

### Comparison of feeding strategies

An overview and direct comparison of the process strategies in the high sugar manually pulsed feeding and the reduced sugar continuous feeding are provided in Table 2. As mentioned above, in the reduced sugar feed less sugar is applied to the process in general and the carbon source utilization is by far more efficient, as less sugar is left unused in the medium.

Regarding the product formation and productivities on glucose, the constant feeding turned out to be 28% more beneficial on cell mass growth and 19% on lipid titer. The total glucose consumption (+ 19%) and cell mass (+ 26%) and lipid (+ 20%) production rates increased compared to pulsed feeding, indicating more favorable production conditions for *S. podzolica* during constant feed. Similar findings on constant feed were observed with *Rhodosporidium toruloides* [14, 29] and *Rhodosporidiobolus fluvialis* [16]*.*

However, the oxidation of glucose to the organic acid gluconic acid is more efficient during pulsed feeding, which is due to the higher glucose excess. As shown by Qian et al. [23], *S. podzolica*’s GA production was greatly enhanced by high carbon source concentration of 150 g/L. The same circumstances are reported in case of high sugar concentration stimulated citric acid production of *Yarrowia lipolytica* and *Rhodotorula glutinis* [18, 30]*.* In the reduced sugar process in this study 48% less GA was produced compared to the high sugar process (Table 2). Considering the improved lipid production in the reduced sugar process, clearly the carbon flux seems to be shifted to lipid accumulation in *S. podzolica* when applying a lower sugar excess. If a co-production of intracellular lipids and extracellular GA is desired, a high sugar excess is recommended, which results, however, in a considerable wasting of carbon source in the cultivation broth [23]. When aiming for target-oriented lipid accumulation, a controlled feeding with constant sugar excess is more suitable, as shown in this study.

The comparison of feeding strategies on xylose provides a different context. Here, product formation is more favored when higher sugar concentrations are pulsed-fed compared to constant feeding. Admittedly, the differences in productivities are rather low and the total lipid productivity rates are even equal (Table 2). By considering the notably reduced applied xylose amount and more efficiently metabolized xylose amount during constant feeding, still the latter should be chosen for a more economical lipid production. In a broader context, a continuous feed with low dilution rates is also more favorable regarding the use of sustainable carbon rich waste products, such as lignocellulosic hydrolytes, crude glycerol or other waste products, as substrate, since such waste products often contain a considerable amount of toxic compounds [16, 24, 31, 32]. Cell proliferation and product formation can be severely compromised upon high concentration of such waste products in a batch or a high sugar excess cultivation. By applying continuously low concentration in a constant feed, this can be overcome and the toxicity stress to the cells can be reduced [16, 33].

By looking at the produced CO_2_ amount it is immediately apparent, that during continuous feeding with both sugars more CO_2_ was emitted compared to pulsed feeding (Table 2). The same can be observed for the amount of CO_2_ produced per dry cell mass (Table 2). For glucose this might be explained due to the fact that during pulsed feeding more GA is produced. The oxidation of glucose to GA is CO_2_ neutral [34]. The by-product formation might be a stress-coping strategy of the yeast to circumvent the high sugar excess, since a higher GA production is reported on higher glucose excess, which was already discussed above. For the process on xylose this argument is however weak, since just low concentrations of XA were produced. Here, it is worthwhile to look at the CO_2_ emission plots over time (Additional file 1: Figure S3). The CO_2_ curves are smoother on continuous feeding of both sugars (Additional file 1: Figure S3a and c), compared to the pulsed fed sugar (Additional file 1: Figure S3b and d), where every 24 h the CO_2_ production is interfered as a feed was applied. Consequently, during continuous feeding the respiration of the cells is less impaired by nutrient supply than during pulsed feeding.

### Comparison of *S. podzolica* cultivation on glucose and xylose

To ensure sustainability and economic feasibility for microbial lipids produced by yeasts, abundant and cheap lignocellulosic plant biomass became of interest as substrate. The main carbohydrates of hydrolysates of lignocellulosic biomass are composed of glucose and xylose [19]. For that reason, the cultivation of *S. podzolica* was optimized on both sugars in this study. It was observed, that total lipid productivities and yields were similar on both carbon sources (Table 2). The volumetric consumption and production rates differ, however, during the cultivation, as presented in Fig. 5. The maximum consumption rate on glucose is higher and lasts longer compared to maximum xylose consumption rate, which indicates glucose as the more efficient and favorable carbon source, as it is the case for most yeasts compared to xylose [35]. Interestingly, although the cell mass production rate on xylose did not reach as a high value as on glucose, it stayed stable at maximum for 14.8 h during the batch phase. Additionally, in the later continuous fed-batch phase the volumetric cell mass production rate on xylose was higher than on glucose. This indicates a similar ability of *S. podzolica* to produce cell mass on both sugars. Also, volumetric lipid production rates are highly comparable. On glucose higher values are reached, however, at the end more efficient productivities are achieved upon xylose. In other studies, with *Candida curvata* (syn. *Cryptococcus curvatus*, *Cutaneotrichosporon oleaginosum*) maximum lipid accumulation occurred in batch culture with xylose as carbon source [36]. For *Lipomyces starkeyi* higher lipid contents were reached on xylose, yet on glucose a higher cell mass concentration was produced [37].

Considering the CO_2_ emission in Additional file 1: Figure S3a for glucose and Additional file 1: Figure S3c for xylose both during continuous feed, both plots differ remarkably. On glucose the CO_2_ emission resembles a parabolic curve, on xylose, however, the CO_2_ emission is more or less stable just below 1.5 mg from the second cultivation day until the end. This can be explained with the different applied glucose and xylose concentration as reported in (Table 5). For cultivation on glucose the starting concentration in the batch phase was higher than the initial xylose concentration, vice versa during fed batch less glucose and more xylose was added. In total a higher total CO_2_ production was observed on xylose with 118.90 g compared to 109.79 g total CO_2_ on glucose, which is 54.08% (Fig. 6b) of total applied xylose channelled to CO_2_ conversion and 46.8% (Fig. 6a) of total glucose metabolized to CO_2_, respectively. For instance, the overall lower carbon to CO_2_ conversion on glucose in contrast to xylose may also be due to the high GA production. GA is the organic acid formed by the oxidation of the first carbon of glucose [34]. Hence, 8.38% (Fig. 6a) of total glucose were channelled to GA production, whereas just 0.41% (Fig. 6b) of total xylose were used to produced XA. Thus, the glucose oxidizing ability of *S. podzolica* is by far more efficient than the xylose oxidation, as can be witnessed by produced amount of and productivity rates (Table 2; Fig. 5).

Nevertheless, both organic acids are of high value. GA is a noncorrosive, nonvolatile, nontoxic, mild carboxylic acid [34]. It can be found abundantly in plants, honey and wine. It is categorized GRAS status and is listed as a generally permitted food additive (E 574) and therefore highly used for food manufacturing [34]. Apart from that, it is regarded as a bulk chemical in the textile, pharmaceutical, and construction industries [38]. Microbial conversion of glucose to GA has also been reported in by *Aspergillus niger*, *Penicillium*, and bacterial species such as *Pseudomonas ovalis*, *Acetobacter*, and *Gluconobacter oxydans* [34]. Admittedly, GA production is just possible with glucose as substrate, which propagates the competition with food purposes. Therefore, the oxidation product of the non-edible xylose XA can substitute GA in some aspects. For instance, XA can be applied for food, pharmaceutical, and agricultural purposes [39], it can be used as sustainable solvent and biocatalyst [40] or as from waste biomass derived polymer precursor [41]. In literature microbial conversion of xylose to XA is reported by *Kluyveromyces lactis*, *Aspergillus niger, Gluconobacter oxydans,* or recombinant *Saccharomyces cerevisiae* and *Escherichia coli* [39, 41, 42]. The XA production ability of *S. podzolica* was to our knowledge never described before. Accordingly, we are the first to report this property of this unconventional oleaginous yeast. Despite the fact, that *S. podzolica*’s XA production rates are rather low, there is potential in that strain to optimize the cultivation process in the purpose to increase the XA production.

## Conclusions

The aim of this study was to provide an optimized cultivation process of *S. podzolica* for lipid production. Adapting temperature and pH improved the produced lipid content by 40%. Furthermore, the intended reduction of applied sugar amount was achieved by establishing a new two-phase process comprising a batch and automated, continuous fed-batch strategy with about 10 g/L excess sugar in the latter phase. Compared to the former pulsed feeding strategy with high carbon source excess, ~ 41% of total glucose and ~ 26% of total xylose could be saved. Using glucose, the reduced sugar cultivation with continuous carbon source supply led to 28% higher cell mass growth and 19% increase of lipid titer. Using xylose, the by-product xylonic acid was detected and identified for the first time as being produced by *S. podzolica.* In conclusion, we were able to optimize the cultivation process for *S. podzolica* with regards to sustainable and economical aspects, since we could reduce the applied sugar amount and avoid high wastage during cultivation without losing productivities. On that account we could establish an automated feeding strategy, which on industrial level could save costs. Finally, we further emphasize the potential of this unconventional oleaginous yeast *S. podzolica* as an interesting production strain for biotechnological purposes. Since renewable lignocellulosic biomass is composed of mixed complex carbohydrates, in future studies, the efficiency of mixed sugars and lignocellulosic hydrolysates will be addressed.

## Materials and methods

### Yeast strain

The yeast strain, which is subject of the study, was first described as *Cryptococcus podzolicus* DSM 27192 by Schulze et al. [15] and deposited at the DSMZ culture collection (Deutsche Sammlung von Mikroorganismen und Zellkulturen; Braunschweig; Germany). After genome sequencing and annotation, the strain was phylogenetically reclassified to *Saitozyma podzolica* DSM 27192 [43].

### Chemicals and cultivation media

All utilized chemicals were obtained from Carl Roth GmbH & Co. KG (Karlsruhe; Germany) or Sigma-Aldrich Chemie GmbH (Taufkirchen; Germany) if not stated otherwise.

The yeast strain was reactivated from cryo preservation at − 80 °C on YM agar (3 g/L yeast extract, 3 g/L malt extract, 5 g/L peptone, 20 g/L agar, pH 7, sterile supplemented with 10 g/L glucose after autoclaving). Mineral salt media for cultivation and lipid production contained a phosphate buffer system (8.99 g/L KH_2_PO_4_ and 0.12 g/L Na_2_HPO_4_ × 2 H_2_O), 0.1 g/L sodium citrate × 2 H_2_O, 0.1 g/L yeast extract, 0.2 g/L MgSO_4_ × 7 H_2_O, 4.72 g/L (NH_4_)_2_SO_4_. After autoclaving 2% (v/v) of sterile trace elements solution with 4 g/L CaCl_2_ × 2 H_2_O, 0.55 g/L FeSO_4_ × 7 H_2_O, 0.475 g/L citric acid, 0.1 g/L ZnSO_4_ × 7 H_2_O, 0.076 g/L MnSO_4_ × H_2_O, 100 μl/L 18 M H_2_SO_4_ and 2% (v/v) of sterile salts solution comprising 20 g/L MgSO_4_ × 7 H_2_O and 10 g/L yeast extract were added. Additionally, glucose or xylose was sterile supplemented.

### Cultivation in shake flasks

For the first pre-culture the yeast cells were scratched from YM agar and transferred into 50 mL mineral salt medium in conical shake flasks and incubated at 130 rpm and 20 °C for 24 h. Second pre-culture was inoculated from first pre-culture to an OD_600nm_ of ~ 1.0 in 200 mL medium and cultivated at the same parameters for 24 h. Main cultures were performed in 2-L conical shake flasks in 400 mL cultivation volume. The cultures were inoculated from second pre-culture to an OD_600nm_ of ~ 1.0 and cultivated at 130 rpm for 96 h. The amount of sugar was daily determined and restocked to 90 g/L. Additionally, a manual feed of 2% (v/v) sterile trace elements and 2% (v/v) of sterile salts solution were added daily. The cultivations were sampled and analyzed for CDW, glucose consumption and lipid content. All experiment set-ups were performed in triplicates.

### Cultivation in bioreactors

For bioreactor cultivations the pre-cultures were conducted in the same way as described for shake-flask cultivation. Main culture was operated in duplicates in 2.5-L glass vessel bioreactors (Infors HT, Bottmingen, Switzerland; Minifors fermentor) with 1.2 L mineral salt medium with initial OD_600nm_ of ~ 1.0. The applied parameters were pH 5 (before optimization) or pH 4 (optimized), the temperature of 20 °C (before optimization) or 22.5 °C (optimized), 600 rpm and 1 vvm aeration rate for 96–144 h. The pH was automatically controlled by addition of 4 M H_3_PO_4_ and 4 M NaOH. Foam formation was detected by a foam probe (Infors HT, Bottmingen, Switzerland) and the anti-foaming agent Contraspum A 4050 HAC (Zschimmer und Schwarz GmbH und Co KG, Lahnstein, Germany) was automatically supplied to prevent foaming. Exhaust gas was monitored with the online exhaust gas analyzers BlueVary controlled by the BlueVis 4.0 Software (BlueSens gas sensor GmbH, Herten, Germany). Yeasts growth and production was tracked by daily sampling and analysis of CDW, carbon source and ammonia consumption and lipid and organic acid content.

#### Manual daily sugar feed in standard process

The initial sugar concentration was 50 g/L. Every 24 h a manual feed of 2% (v/v) sterile trace elements, 2% (v/v) sterile salts solution and carbon source supply to 90 g/L of glucose or 60 g/L of xylose was implemented after determining the consumed sugar amount. The daily and the total applied amount of carbon source is illustrated in Table 3.

**Table 3:** Sugar amount used for the high sugar excess standard process with manual daily sugar restock

	Sugar amount applied in batch phase [g] (c_end_ [g/L]^a^)	Applied sugar amount for daily restock [g]					Total applied sugar [g]
		24 h	48 h	72 h	96 h	120 h	
Glucose	60 (50)	71.5	46	37.5	–	54	269
Xylose	60 (50)	23	46	30	23.5	21.5	204

^a^Initial cultivation volume was 1.2 L

#### Manual daily sugar feed in adjusted process

To develop a reduced sugar excess process, data of five independent cultivation experiments were used to calculate the average sugar consumption to establish a new feeding strategy. The cultivation method was adapted to the calculated consumption rate resulting in a two-phase process with initial batch fermentation for the first 48 h merging into a daily fed-batch strategy aiming for a carbon source excess of approximately 10 g/L at the lowest point for four more days. The daily manual feeds included 2% (v/v) sterile trace elements, 2% (v/v) sterile salts solution and the respective sugar amount, which is reported in Table 4.

**Table 4:** Sugar amount used for the reduced sugar excess adjusted process with pulsed daily sugar restock

	Sugar amount applied in batch phase [g] (c_end_ [g/L]^a^)	Applied sugar amount for manual fed batch [g]				Total applied sugar [g]
		1st day	2nd day	3rd day	4th day	
Glucose	71 (59.2)	31.5	24.5	21	21	169
Xylose	49 (40.8)	24.5	19	13	14.5	120

^a^Initial cultivation volume was 1.2 L

#### Automated continuous feed

To establish an automatic supply of carbon source, trace elements and salts solution during the fed-batch phase the feed solutions were prepared containing 50% (v/v) of 500 g/L concentrated stock solution of the respective sugar, 25% (v/v) of sterile trace elements solution and 25% (v/v) of sterile salts solution. Operated sugar amount for the feed solutions are displayed in Table 5. The density ρ of glucose and xylose feed was determined and consequently daily feed rates were calculated in g/h (Table 5). The continuous feed was enabled by external peristaltic pumps (Watson-Marlow GmbH, Rommerskirchen, Germany), regulated by the weight reduction of the feed bottle located on a laboratory balance (OHAUS Adventurer Pro AV4102C, OHAUS Europe GmbH, Nänikon, Switzerland) and controlled by LabVIEW software (LabVIEW2016, National Instruments, Austin, TX, USA). The resulted daily dilution rates D are presented in Table 5.

**Table 5:** Sugar amount used for automated feed cultivations and daily pumping rates of the feeding solutions

	Sugar amount applied in batch phase [g] (c_end_ [g/L] ^a^)	Sugar amount applied for continuous fed batch [g]	Total applied sugar [g]	Daily feed rates [g/h] (dilution rate D [1/h])			
				1st day	2nd day	3rd day	4th day
Glucose	71 (59.2)	89	160	4.20 (0.0035)	4.19 (0.0035)	4.19 (0.0034)	3.45 (0.0027)
Xylose	49 (40.8)	101	150	5.40 (0.0044)	4.20 (0.0033)	4.19 (0.0032)	4.22 (0.0032)

^a^Initial cultivation volume was 1.2 L

### Cell dry weight (CDW) determination

Cell dry weight (CDW) analysis was performed gravimetrically for each sample in duplicates. In pre-dried and pre-weighted 1.5-mL reaction tubes 1 mL cultivation broth was provided and centrifuged at 20,000×*g* for 3 min. The supernatant was used for determination of carbon source, organic acid and ammonium concentration. The cell pellet was washed with 0.8 mL physiological saline (0.9% w/v NaCl) at 20,000×*g* for 3 min. The saline was withdrawn and the pellet was dried for 48 h at 60 °C and weighed with a precision balance.

### Carbon source quantification

Yeast cultivations were performed on glucose or xylose. Glucose consumption was determined enzymatically using the UV-method at 340 nm of the d-glucose test kit from R-Biopharm (Art. No. 10716251035, R-Biopharm AG, Darmstadt, Germany). All from manufacturer-instructed volumes were minimized to one-third and besides the manufacturer’s protocol was followed. Samples were diluted with 0.9% w/v NaCl to appropriate concentration before detection.

Xylose was measured with a standard HPLC apparatus (Agilent 1100 Series, Agilent Technologies Deutschland GmbH, Böblingen, Germany) equipped with a Rezex ROA organic acid H+ (8%) guard column (8 µm, 50 × 7.8 mm) (Phenomenex Inc., Aschaffenburg, Germany) followed by a Rezex ROA organic acid H+ (8%) column (8 µm, 300 × 7.8 mm) (Art. No. 00H-0138-K0, Phenomenex Inc., Aschaffenburg, Germany) under isocratic conditions at 50 °C column temperature for 20 min with 5 mM H_2_SO_4_ as mobile phase at a constant flow rate of 0.5 mL/min. The injection volume was 10 µL. The detection was enabled via a refractive index detector (Agilent 1200 series, Agilent Technologies Deutschland GmbH, Böblingen, Germany). For detection, cultivation broth was diluted in 5 mM H_2_SO_4_ to appropriate concentrations.

### Organic acid analysis via HPLC

For sample preparation cultivation broth was diluted in 20 mM KH_2_PO_4_ pH 2.5 to appropriate concentrations for detection. Gluconic acid and xylonic acid were quantified with a standard HPLC device (Agilent 1100 Series, Agilent Technologies Deutschland GmbH, Böblingen, Germany) using the reversed phase column Synergi™ 4 μm Fusion-RP 80 Å (150 × 4.6 mm) (Art. No. 00F-4424-E0, Phenomenex Inc., Aschaffenburg, Germany). Compounds were separated in a gradient mobile phase system of 20 mM KH_2_PO_4_ pH 2.5 (A) and 100% methanol (B) with a flow rate of 1 mL/min. The elution conditions of the gradient were 0–0.5 min 100% eluent A, 0.5–10 min formation of the gradient of eluent B from 0 to 10%, 10–12 min decrease of eluent B back to 0%, 12–14 min reconditioning step of the column to 100% eluent A. The injection volume was 10 µL and the temperature of the column oven set to 30 °C. The detection was performed with a UV detector at a wavelength of 220 nm. To quantify and identify the peaks of both acids, calibration curves of analytical standards were performed with d-Xylonic acid (Art. No. 73671-100MG; Sigma Aldrich; Taufkirchen; Germany) and d-Gluconic acid (Art. No. 64188-100MG; Sigma Aldrich; Taufkirchen; Germany).

### Ammonia determination

Ammonium nitrogen was analyzed photometrically using the Spectroquant kit (1.14752.0001, Merck KGaA, Darmstadt, Germany). For the assay requested volumes were down-scaled to 300 μL per sample and measured in microtiter plates in duplicates following the manufacturer’s instruction procedure.

### Indirect lipid quantification via derivatization to FAME

Indirect quantification of produced lipids was conducted by direct transesterification of yeast cell mass to fatty acid methyl esters (FAMEs) as described in Schulze et al. [15] and further optimized in Gorte et al. [44]. 15 mL of fresh cell mass was collected daily from the cultivation broth and washed with 0.9% w/v NaCl at 4800×*g* for 5 min. The pellet was freeze-dried for 24 h at − 30 °C and 0.370 mbar using the BETA 1–8 freeze dryer (Christ, Osterode am Harz, Germany). 20–30 mg of freeze-dried cell mass was applied in a glass tubes and a subsequent acidic transesterification was performed using a two-phase system. The hexane phase contained 0.5 mL internal standard consisting of 2 mg/mL heptadecanoic acid in hexane and 1.5 mL pure hexane. The second phase had an equal volume of 2 mL 15% H_2_SO_4_ (reaction’s catalyst) in methanol. The mixtures were incubated for 2 h at 100 °C and 1000 rpm in a thermo-shaker (Universal Labortechnik, Leipzig, Germany). All samples were additionally mixed every 30 min by vortexing. To stop the reaction, the tubes were placed on ice for 10 min. To improve phase separation, 1 mL distilled water was added. The upper hexane phase containing FAMEs was used for GC analysis.

### GC analysis of fatty acid methyl esters (FAMEs)

The quantitative and qualitative analysis of FAMEs was performed via gas chromatography (GC) using the 6890N Network GC-System (Agilent Technologies Deutschland GmbH; Waldbronn; Germany). The device was coupled with a DB-Wax column (30 m × 0.25 mm) (Art. No. 122–7032; Agilent Technologies Deutschland GmbH, Böblingen, Germany) and the detection was performed with a flame ionization detector under 1.083 bar working pressure. 1 µL of sample was injected at the initial temperature of 40 °C. The separation was achieved by a temperature gradient from 40 to 250 °C with a rate of 8 °C/min and was kept for 10 min at 250 °C. To identify and quantify the FAMEs, the RM3 FAME Mix standard (Art. No. 07256-1AMP; Sigma Aldrich; Taufkirchen; Germany) was used.

### Used approximation for carbon balance

To give an assumption about the carbon conversion in the process, absolute mass of applied sugar, by yeasts produced CO_2_, cell mass (CDW), lipids, by-products (GA or XA, respectively) and excess sugar at process end was calculated and based on the molecular weight of carbon. To do so, in case of cell mass the empirical chemical formula C_6_H_10_O_3_N for baker’s yeast cell mass [45] was used after subtraction of the lipid content, since lipids are intracellular products and therefore also weighted with the CDW determination method. Thus the approximation of carbon converted to lipid-free cell mass was calculated in case of CDW.

### Statistical analysis

#### Mean of levels comparison

One-way ANOVA followed by post hoc Tukey test using *p*-value < 0.05 were performed with the Origin Software [version 2019 (9.6)] to identify significant differences in the means of values.

#### Fit of data

Sugar consumption, cell mass production, lipid titer and organic acid concentration were fit using Origin Software [version 2019 (9.6)] performing the nonlinear curve fit with logistic model after Levenberg–Marquardt method with four parameters upon the equation:

$${y}=\frac{{A}_{1}-{A}_{2}}{{1+(\frac{x}{{x}_{0}})}^{p}}+{A}_{2}.$$

The parameter *A*_1_ indicates the initial data value at *x* = 0. *A*_2_ is the final data value. *x*_0_ is half way between the two limiting values *A*_1_ and *A*_2_ and indicates the mid-range (50%). *p* is the slope factor. The Levenberg–Marquardt regression solves nonlinear least-squares problems by converging from a wide range of initial guess values of parameters [46]. It is often used as logistic dose response in science and engineering.

## Supplementary information

**Additional file 1: Figure S1.** Investigation of the optimal temperature for lipid production of *S. podzolica* in shake flasks. 18, 20, 22, 25 and 27 °C were tested over a 96 h cultivation. The data were normalized to the highest lipid titer [g/L] value. The error bars result from the standard deviation of experimental set-ups in triplicates each. **Figure S2.** Illustration of produced lipids by *S. podzolica* in bioreactors at 22 °C at three different pH of 4,5 and 6 in 96 h process time. The data were normalized to the highest lipid titer [g/L] value. The error bars result from the standard deviation of duplicate experimental set-ups. **Figure S3.** CO_2_ emission of *S. podzolica* at different cultivation modes over time. (a) Automated continuous feed process on glucose. (b) Daily pulsed glucose restock to 90 g/L. (c) Automated continuous feed process on xylose. (d) Daily pulsed xylose restock to 60 g/L.

## Data Availability

All data generated or analyzed during this study are included in this published article and its additional file.

## Abbreviations

CFS: Calculated feed strategy
CDW: Cell dry weight
FAME: Fatty acid methyl ester
HPLC: High performance liquid chromatography
GC: Gas chromatography
FID: Flame ionization detector
GA: Gluconic acid
XA: Xylonic acid
