# Supplementary material for: Optimization of carbon source efficiency for lipid production with the oleaginous yeast Saitozyma podzolica DSM 27192 applying automated continuous feeding
Source: Biotechnol Biofuels. 2020 Nov 2;13:181. doi: 10.1186/s13068-020-01824-7 (PMC7607716; doi:10.1186/s13068-020-01824-7)
Supplement: Supplementary file 1 — Additional file 1: Figure S1. Investigation of the optimal temperature for lipid production of S. podzolica in shake flasks. 18, 20, 22, 25 and 27 °C were tested over a 96 h cultivation. The data were normalized to the highest lipid titer [g/L] value. The error bars result from the standard deviation of experimental set-ups in triplicates each. Figure S2. Illustration of produced lipids by S. podzolica in bioreactors at 22 °C at three different pH of 4,5 and 6 in 96 h process time. The data were normalized to the highest lipid titer [g/L] value. The error bars result from the standard deviation of duplicate experimental set-ups. Figure S3. CO2 emission of S. podzolica at different cultivation modes over time. (a) Automated continuous feed process on glucose. (b) Daily pulsed glucose restock to 90 g/L. (c) Automated continuous feed process on xylose. (d) Daily pulsed xylose restock to 60 g/L. [file 13068_2020_1824_MOESM1_ESM.pdf]

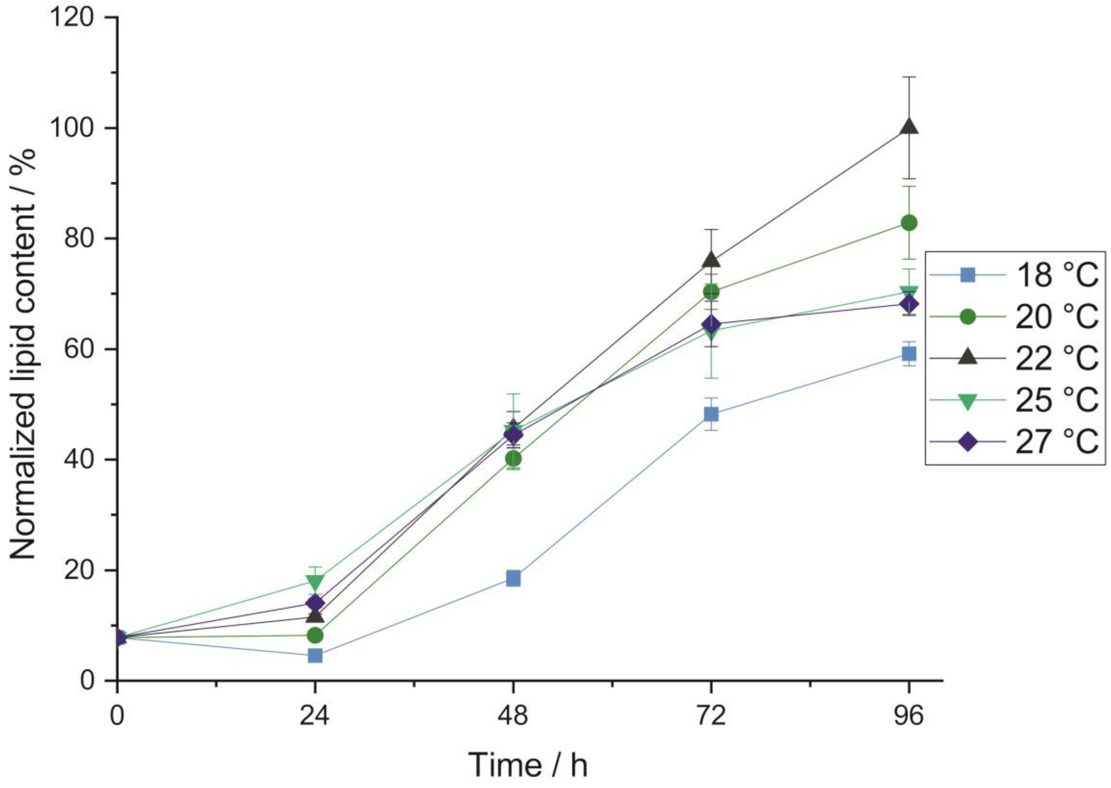

Figure S1: Investigation of the optimal temperature for lipid production of *S. podzolica* in shake flasks. 18, 20, 22, 25 and 27 °C were tested over a 96 h cultivation. The data were normalized to the highest lipid concentration [g/L] value. The error bars result from the standard deviation of experimental set-ups in triplicates each.

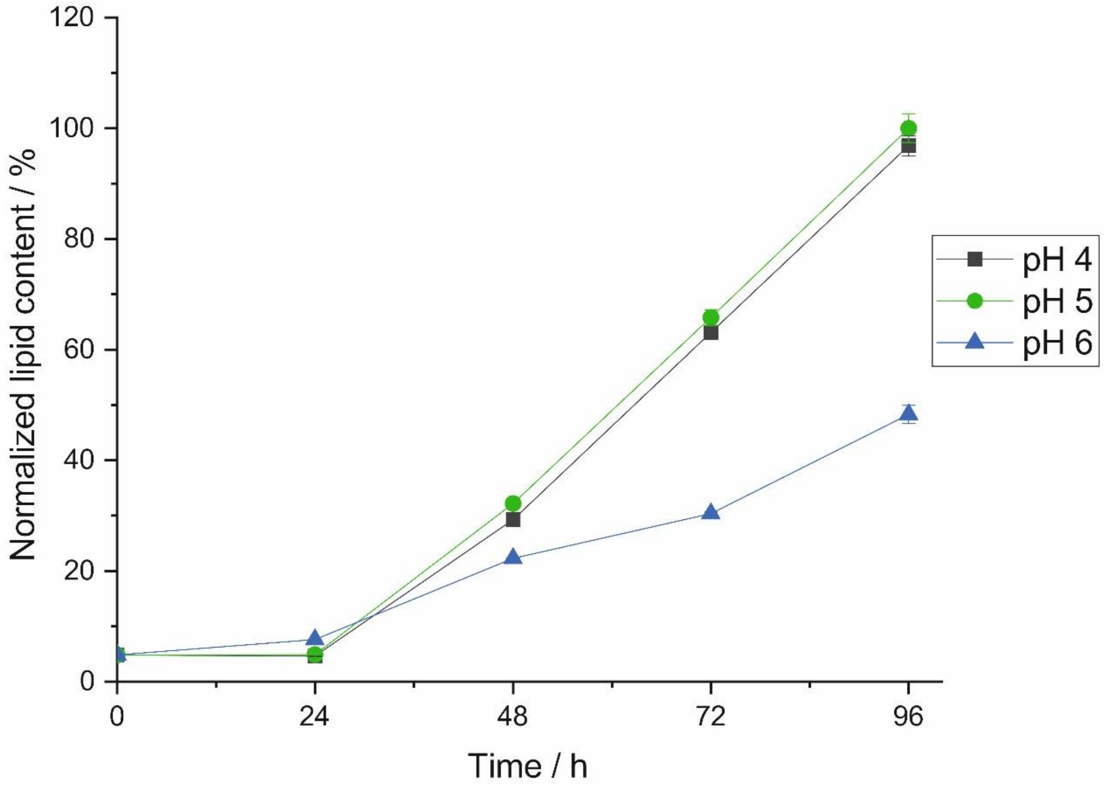

Figure S2: Illustration of produced lipids by *S. podzolica* in bioreactors at 22 °C at three different pH of 4,5 and 6 in 96 h process time. The data were normalized to the highest lipid concentration [g/L] value. The error bars result from the standard deviation of duplicate experimental set-ups.

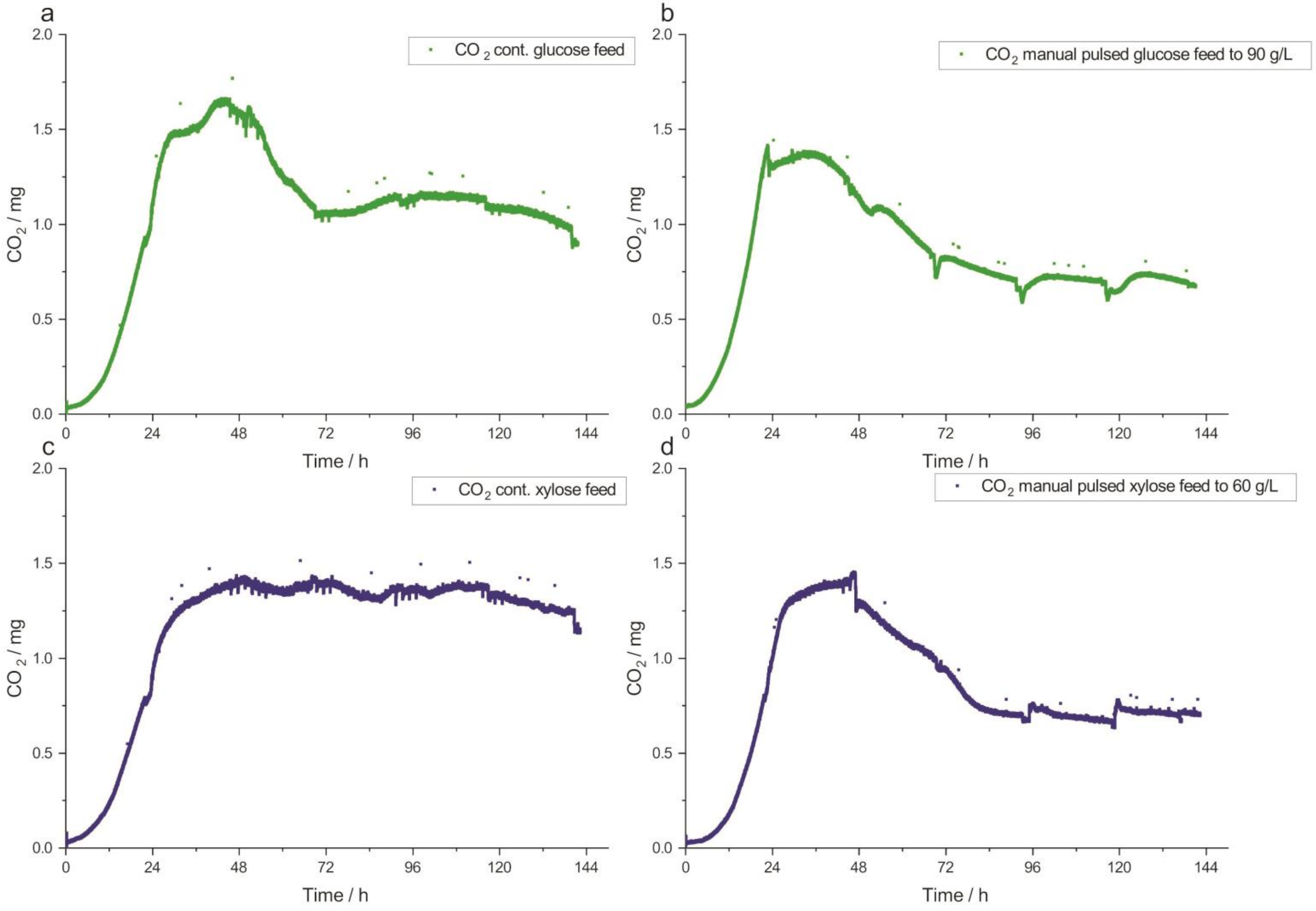

Figure S3: CO_2 emission of *S. podzolica* at different cultivation modes over time. a) automated continuous feed process on glucose. b) daily pulsed glucose restock to 90 g/L. c) automated continuous feed process on xylose. d) daily pulsed xylose restock to 60 g/L.
